# Noninvasive Evaluation of Prolonged‐Release Pirfenidone in Compensated Liver Cirrhosis. ODISEA Study, a Randomised Trial

**DOI:** 10.1111/liv.70131

**Published:** 2025-05-22

**Authors:** Linda E. Muñoz‐Espinosa, Aldo Torre, Laura Cisneros, Iaarah Montalvo, René Malé, Scherezada Mejía, Juan Ramón Aguilar, Javier Lizardi, Jaime Zuñiga‐Noriega, María Eugenia Icaza, Frida Gasca‐Díaz, Larissa Hernández‐Hernández, Paula Cordero‐Pérez, Luis Chi, Lilian Torres, Fátima Rodríguez‐Alvarez, Graciela Tapia, Jorge Luis Poo

**Affiliations:** ^1^ Universidad Autónoma de Nuevo León, “Dr. José E. González” University Hospital Monterrey Mexico; ^2^ Instituto Nacional de Ciencias Médicas y Nutrición Ciudad de México Mexico; ^3^ Christus Muguerza Hospital Monterrey Mexico; ^4^ Liver Clinic Mérida Mexico; ^5^ Digestive and Hepatic Disease Institute Guadalajara Mexico; ^6^ Juarez Hospital Mexico City Mexico; ^7^ Mexican Group for the Study of Liver Diseases (PROMHEPA) Mexico City Mexico; ^8^ Universidad Nacional Autónoma de México Mexico City Mexico

**Keywords:** cirrhosis, fibrosis, prolonged‐release pirfenidone

## Abstract

**Background:**

Advanced liver fibrosis (ALF) predicts an adverse prognosis in chronic liver disease. In addition to etiological treatment, a new approach to stop or reverse residual fibrosis is desirable.

**Objective:**

To assess the efficacy and safety of prolonged‐release pirfenidone (PR‐PFD) versus placebo in compensated cirrhosis.

**Methods:**

180 patients with ALF (F4) were randomly assigned to: placebo, 1200 mg/d, and 1800 mg/d PR‐PFD, plus standardised care, for 24mo. Frequency of lab tests: (3mo), liver stiffness measurement (LSM), FibroTest, ultrasound (US) (6mo), and endoscopy (annually).

**Results:**

Fibrosis evolution estimated from LSM was significantly lower only in the 1200 compared to placebo and 1800 groups (24.2 ± 2.4 vs. 15.4 ± 2.4; 27.6 ± 2.4 vs. 24.6 ± 2.4; 24.4 ± 2.3 vs. 23.3 ± 2.3 kPa, respectively, *p* < 0.001), in intergroup analysis, meeting the primary endpoint. Fibrotest was significantly lower only in the 1200 mg/d group, compared to baseline values (0.86 ± 0.02 vs. 0.83 ± 0.02 units, *p* < 0.001). Liver function test (LFT's) also improved as well as Model for End‐Stage Liver Disease (MELD) score and quality of life (QoL). Decompensations occurred in 19 patients: 12 ascites (more frequent in placebo, *p* = 0.003), 5 variceal bleeding, 4 encephalopathies, 4 hepatocarcinomas. Adverse events were mainly mild gastrointestinal (*n* = 35, 48 and 46, *p* = 0.010) and cutaneous (*n* = 12, 15, and 22, *p* = 0.0001) in placebo, 1200 and 1800 mg/day, respectively.

**Conclusion:**

PR‐PFD at a dose of 1200 mg significantly decreased non‐invasive liver fibrosis markers at 24 months and induced improvement in LFT's, MELD, and QoL in compensated cirrhosis, without safety concerns.

**Trial Registration:**

ClinicalTrials.gov identifier: NCT01046474

**Summary**

- This is a new treatment approach for advanced liver fibrosis, aiming to stop or reverse the condition.
- Involving 180 patients, the study compared placebo with two doses (1200 and 1800 mg) of a drug called PR‐PFD over 24 months.
- It found that the 1200 mg dose significantly reduced liver fibrosis compared to the placebo, improved liver function, and enhanced quality of life.
- While there were mild side effects, such as digestive and skin issues, they were more common in the drug groups than in the placebo.
- Overall, the 1200 mg dose was effective and safe for improving liver health in patients with compensated cirrhosis.

**Abbreviations**
AEs: adverse events
AILD: autoimmune liver diseases
ARLD: alcohol‐related liver disease
ALF: advanced liver fibrosis
CSPH: clinically significant portal hypertension
DM: diabetes mellitus
FDA: Food and Drug Administration
FPP: fibrosing‐progression profile
FRP: fibrosing‐regression profile
FSP: fibrosing‐stable profile
FT: FibroTest
HCC: hepatocellular carcinoma
HCV: hepatitis C virus
IPF: Idiopathic pulmonary fibrosis
kPa: kilo Pascals
LSM: liver stiffness measurement
MASLD: steatotic liver disease associated with metabolic dysfunction
MELD: Model for End‐Stage Liver Disease
NITs: non invasive tests
PFD: pirfenidone
PHT: portal hypertension
PR‐PFD: prolonged‐release pirfenidone
SAH: systemic arterial hypertension
TE: transient elastography

## 1 Introduction

Over the past five decades, liver cirrhosis has represented a significant and increasing global health burden [1]. According to the World Health Organisation, liver cirrhosis is responsible for a substantial number of deaths worldwide, with an estimated 1.32 million deaths in 2019 alone [2]. In particular, Mexico has been identified as the region of the Americas with the highest prevalence of compensated and decompensated cirrhosis [3], thereby emphasising the urgent need for better treatment strategies [1, 4]. The progression of fibrosis is undoubtedly an important factor in predicting the long‐term prognosis of patients with chronic liver disease (CLD) [5].

The first line of treatment should be the etiological factor of liver cirrhosis. However, some patients continue with persistent fibrosis despite treating or controlling the cause and may benefit from direct antifibrotic therapy, especially those with compensated cirrhosis, as it is difficult to predict “the point of no return.” Pirfenidone (PFD) is recognised for its effectiveness in the treatment of fibrotic conditions in various organs, particularly the lung, and also in kidney, heart, pancreatic, liver, and skin tissues [6, 7]. This multifaceted drug attenuates several proinflammatory cytokines, reduces oxidative damage and apoptosis, inhibits fibroblast activation and may behave as a PPAR α agonist in metabolic dysfunction‐associated steatotic liver disease (MASLD) [8]. Our group previously reported PFD benefits in a non‐controlled clinical trial with 122 cirrhotic patients [9].

Patients who use pirfenidone for several years often experience improved lung function and quality of life, as it helps preserve respiratory function and slow disease progression [7]. Its benefits extend beyond pulmonary fibrosis and thanks to its availability for oral administration and low toxicity profile, we consider it to be promising in the treatment of other fibrotic disorders such as liver fibrosis. Pirfenidone, without a doubt, is a ray of hope for people fighting liver fibrosis, since it offers a therapeutic route that is easy to implement and with promising results according to several experimental [10, 11, 12] and clinical studies [13, 14].

Therefore, we aimed at evaluating whether therapy with a novel prolonged‐release pirfenidone (PR‐PFD) formulation specifically designed to reduce toxicity and achieve constant plasma levels over a long period of time, in combination with standard care, would facilitate the reduction of reliable non‐invasive tests (NITs) for liver fibrosis and offer a more favourable risk–benefit profile than a placebo in patients with compensated liver cirrhosis.

## 2 Methods

This was an experimental, randomised, double‐blind, multicenter, placebo‐controlled clinical study to evaluate the efficacy and safety of PR‐PFD at two different doses over 24 months. Compensated liver cirrhosis status was determined on the basis of clinical, biochemical, ultrasound, and endoscopic findings compatible with chronic damage, and at least two other non‐invasive liver fibrosis methods that confirmed grade 4 fibrosis (F4).

### 2.1 Inclusion and Exclusion Criteria

A total of 223 patients with compensated cirrhosis from seven sites participated.

Recruitment started on June 26, 2015, and the last patient visit was on December 08, 2021. In HCV patients, antiviral treatment and the achievement of SVR for at least one year were required. Similarly, in ARLD patients, at least one year of alcohol abstinence was required. Patients with previous hepatic decompensation, exposure to herbal/alternative medicine, or any hepatotoxic drug were excluded. Also, concomitant systemic infection; prior history of malignancy, hemoglobinopathy, or any disease associated with hemolysis; history of significant renal, cardiac, or pulmonary disease; alpha‐fetoprotein > 100 ng/L; pregnancy; and alcohol or intravenous drug abuse within the previous year were excluded. Patients with large varices without previous haemorrhage received beta blockers and/or variceal band ligation as primary prophylaxis according to Baveno Vll [15].

### 2.2 Study Design and Treatment Regimens

The study was conducted in compliance with the International Good Clinical Practices and the principles of the Declaration of Helsinki. The protocol was approved by local IRBs and the Mexican Drugs Agency (COFEPRIS).

Treatment consisted of 600 mg tablets of PR‐PFD (which is the standard tablet dose available and marketed as Kitoscell LP in Mexico) or identical placebo tablets, taken orally, one tablet in the morning and two tablets at night after meals. The patients were randomly distributed into three groups: Group 1 (G1), placebo tablets (*n* = 60 patients); Group 2 (G2), 1200 mg of PR‐PFD per day (*n* = 60 patients); and Group 3 (G3), 1800 mg of PR‐PFD per day (*n* = 60 patients). All participants received standard care that included nutritional support, quarterly medical evaluation to review laboratory results, and adjust medications.

### 2.3 Concomitant and Alternative Medications

During this study, the use of medications for DM, dyslipidemia, or SAH, beta blockers for variceal haemorrhage prophylaxis, steroids, and immunosuppressants in patients with AILD, at the dose decided by the attending hepatologist, was permitted at stable doses.

### 2.4 Clinical and Laboratory Evaluation

The patients' lab tests were evaluated every three months. Hepatic echography and elastography semiannually, and endoscopic evaluations annually. The patients' somatometric measurements (height and body weight) and frequency of adverse events (AEs) were recorded. The biochemical markers determined after overnight fasting included albumin, prothrombin time, total bilirubin, ALT, AST, ALP, and GGT, measured using an automated biochemistry analyser (Roche/Hitachi, Tokyo, Japan).

For the Fibro Test (FT) evaluation (BioPredictive, Paris France), fresh serum was used, according to the recommended methods [16]. The FIB‐4 score was calculated as follows: FIB‐4 = (age (years) × AST (U/L))/(PLT count (10^9^/L) × (ALT (U/L))^1/2^) [17].

Transient elastography (TE) was performed according to published recommendations using the Fibro‐Scan M probe. Liver stiffness measurements (LSM) were expressed in kilopascals (kPa). Only procedures with at least 10 validated measurements and an interquartile range < 30% of the median were considered reliable. The semi‐quantitative analysis used predetermined cut‐offs for the non‐cirrhotic stages of F0 (0–5 kPa), F1 (> 5–7.1 kPa), F2 (> 7.1–9.5 kPa), and F3 (> 9.5–12.5 kPa). Cirrhotic stages were F4 (> 12.5 kPa) and clinically significant portal hypertension (CSPH > 25 kPa) [18].

In a subgroup of 43 patients (placebo, *n* = 8; 1200 mg, *n* = 17; 1800 mg, *n* = 18) plasma levels of PFD were measured in the same patients at each visit, in fasting conditions using an HPLC method with UV detection as previously described by our group [9, 19].

### 2.5 Study End Points

The primary efficacy endpoint was a statistically significant reduction in the fibrosis score (kPa) according to hepatic elastography or FT units. Secondary efficacy endpoints included improvement in ALT and/or AST, albumin, bilirubin, and Child–Pugh and MELD scores. We also evaluated the progression of liver disease according to Baveno VII consensus regarding CSPH [15]. and through the use of validated NITs [20].

Regarding the quality of life assessment, all patients filled out the EuroQol Index survey, including the visual analog scale evaluation, which ranged from 0 (worst imaginable health state) to 100 (best imaginable health state). Measurements in the EuroQol five‐dimension scale [21] and the modified fatigue impact scale (MFIS) were measured every six months [22].

### 2.6 Evaluation and Classification of Fibrosis Outcomes

The fibrosis‐regression profile (FRP) is defined by decreases greater than 10% in FT score or 50% in kPa in LSM at months six, 12, 18, and 24. The fibrosis‐stabilisation profile (FSP) was assessed by stable FT results or kPa measurements (variations lower than 10% or 50%, respectively). The fibrosis‐progression profile (FPP) was reflective of increases greater than 10% or 50% in FT or kPa scores.

### 2.7 Evaluation of Safety Profile

Safety and toxicity were monitored throughout the study (WHO grade modified ACTG graded toxicity scale). When necessary, appropriate medical intervention was provided. PR‐PFD was suspended in any patient who experienced severe clinical (e.g., photosensitivity) or laboratory toxicity (grade 3, modified AIDS Clinical Trials Group graded toxicity scale), until toxicity resumed to baseline values.

### 2.8 Statistical Data Analysis

Statistical evaluation was performed using SPSS software version 26 for Windows. Discrete variables are summarised as counts (percentages) and continuous variables as mean ± SD or ± SEM. Non‐normally distributed variables were log10 transformed for some statistical analyses and for graphical comparisons. In univariate statistical comparisons, the chi‐square test was used for categorical variables, whereas the Student's t‐test or analysis of variance was used for normally distributed continuous variables. For the efficacy analysis, we used an ANCOVA model, with multiple imputation method after verifying that the missing values were randomly distributed.

A paired or unpaired t‐test was used to compare means before and after study medication administration, with Bonferroni correction applied when applicable. Values < 5% were considered significant. As a complementary tool, a repeated measure analysis was executed with a mixed model [23]. A correlation analysis was carried out between the FIB‐4 score and the fibrosis score in kPa. In cases of possible significant relationships between two independent variables, multiple linear regression was applied. For MELD profiles and AE analysis, we used the likelihood ratio test.

Sample size calculation was done with the G power statistical program [24] using F ANOVA for repeated measures between factors, and the following assumptions: (a) alpha error of 5%; (b) an accepted beta error of 20% (power = 80%); (c) number of groups = three; (d) number of measurements = five; and (e) effect size = 0.125. The final number of patients required to find a significant difference was 99 patients. The number needed to treat (NNT) was also calculated [25]. It was found that 3.9 patients were required to achieve a potency of 0.901 at the 24‐month evaluation.

### 2.9 Funding Source

Mexican group for the study of liver diseases (PROMHEPA) was involved in study design, conduct, and data analysis. Identical placebo and study‐drug medications (1200 and 1800 mg) were provided by Grupo Medifarma, México.

## 3 Results

**FIGURE 1:**
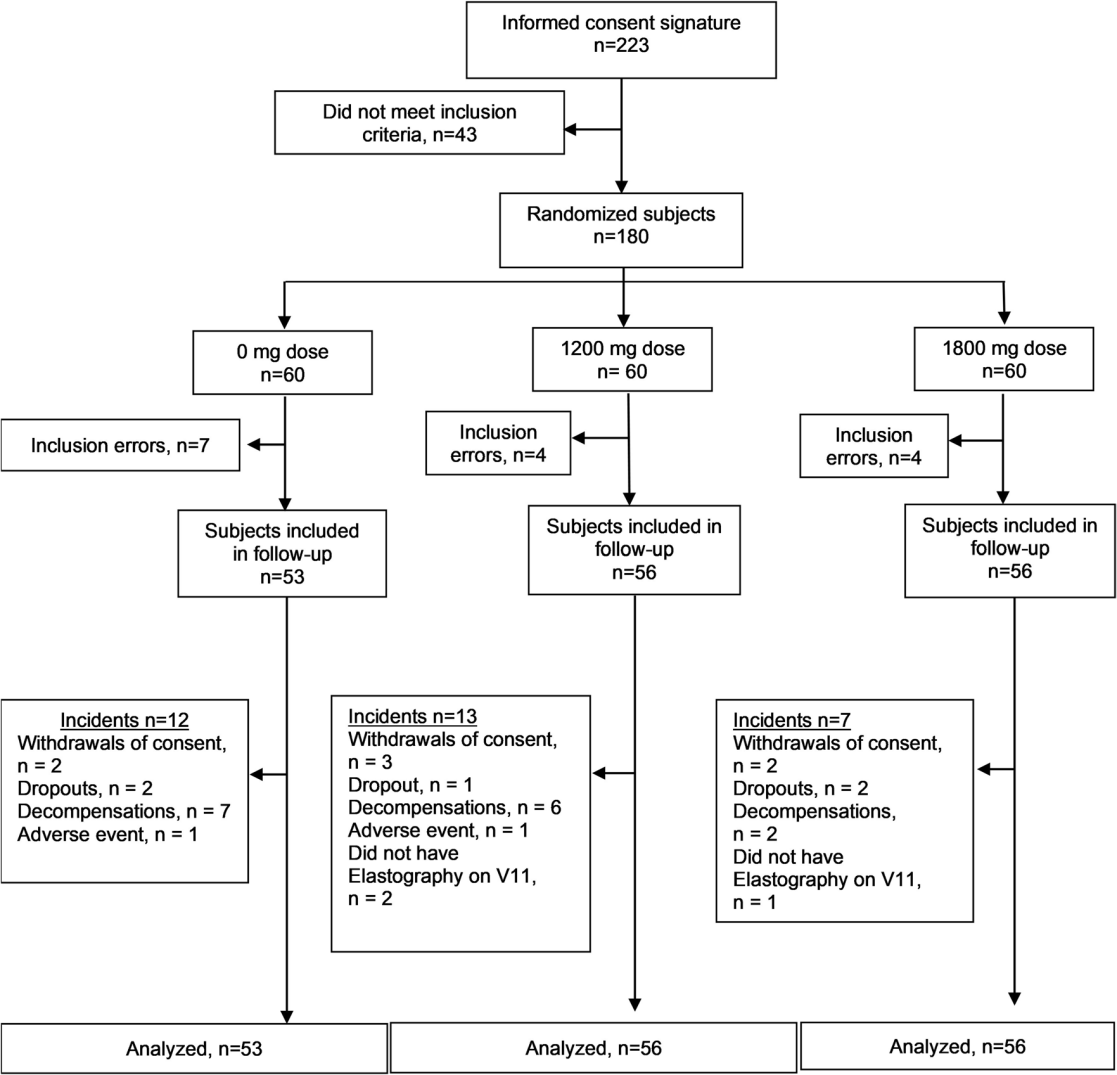
ODISEA study population flow chart.

The ODISEA study flowchart, according to CONSERT recommendations for the total study population, is shown in Figure 1.

The study population comprised 180 patients, 110 women. The etiologies were hepatitis C virus related (HCV) in 71 (39.4%), MASLD in 67 (37.2%), alcohol‐associated/related liver disease (ARLD) in 20 (11.2%), autoimmune liver disease (AILD) in 15 (8.3%), and cryptogenic in seven (3.9%) patients. All patients with HCV, previously treated for their viral disease, presented with cirrhosis.

Concomitant disease was detected in 144 patients (80%), including diabetes mellitus (DM, *n* = 61, 33.9%), systemic arterial hypertension (SAH, *n* = 84, 46.7%), dyslipidemia (*n* = 28, 15.6%), and obesity (*n* = 99, 55.0%).

Only 165 participants were eligible for the follow‐up study and considered candidates for the intention‐to‐treat (ITT) analysis (placebo *n* = 53; 1200 mg, *n* = 56; 1800 mg, *n* = 56). Because our study took place partially during the COVID‐19 pandemic and patients' own decisions to remain active during the 24‐month study, only 105 patients were eligible for the per‐protocol (PP) analysis (placebo *n* = 33; 1200 mg, *n* = 35; 1800 mg, *n* = 37), which meets our intended target population.

The baseline demographic characteristics, aetiologies, Child‐Pugh and MELD scores, and cirrhosis stage categories for the three study groups are described in Table 1 and the baseline biochemical findings in Table 2. The three groups of patients were comparable in basal conditions.

**TABLE 1:** Demographics, cirrhosis aetiology, Child‐Pugh, and MELD scores at baseline of ITT patients (*n* = 165).

Parameter	Placebo *n* = 53	1200 mg *n* = 56	1800 mg *n* = 56	Statistical test	*p*
Age (y)	57 ± 9	59 ± 10	59 ± 9	*F*, 0.820	0.442
Gender female	68%	45%	66%	Chi square 7.640	0.022
Cirrhosis aetiology					
HCV MASLD AILD ALD Cryptogenic	34.0% 49.1% 7.5% 5.7% 3.7%	44.6% 30.4% 3.6% 16.1% 5.3%	39.3% 33.9% 10.7% 12.5% 3.6%	Chi square 4.868	0.301
Child‐Pugh score	5.24 ± 0.09	5.26 ± 0.09	5.40 ± 0.09	*F*, 0.877	0.418
Child‐Pugh class					
A B	96.2% 3.8%	92.9% 7.1%	89.1% 10.9%	Chi square 2.016	0.350
MELD score	9.8 ± 0.4	9.7 ± 0.4	9.4 ± 0.4	*F*, 0.285	0.753
MELD group					
≤ 9 10–19 ≥ 20	53.8% 46.2% 0	51.8% 46.4% 1.8%	58.2% 41.8% 0	Chi square 2.517	0.642
Cirrhosis stage					
1 2	30.0% 70.0%	41.8% 58.2%	37.0% 63.0%	Chi square 3.715	0.446

*Note:* Values are given as mean ± SD or percentages.

**TABLE 2:** Biochemical scores at baseline of ITT patients (*n* = 165).

Parameter	Placebo *n* = 53	1200 mg *n* = 56	1800 mg *n* = 56	Statistical test	*p*
Haemoglobin (g/dL)	14.3 ± 0.25	14.4 ± 0.24	14.5 ± 0.25	*F*, 0.156	0.855
Leucocytes (×10^3^)	4.8 ± 0.21	4.7 ± 0.20	4.6 ± 0.20	*F*, 0.406	0.667
Platelets (×10^3^)	112.4 ± 7.8	121.7 ± 7.7	122.4 ± 7.6	*F*, 0.526	0.592
Bilirubin (mg/dL)	1.01 ± 0.09	0.90 ± 0.09	1.06 ± 0.09	*F*, 0.840	0.434
Albumin (mg/dL)	4.04 ± 0.07	4.15 ± 0.07	4.15 ± 0.07	*F*, 0.925	0.399
Prothrombin time (INR)	1.33 ± 0.05	1.31 ± 0.04	1.28 ± 0.04	*F*, 0.333	0.718
ALT (IU/L)	49.1 ± 4.9	43.5 ± 4.7	42.1 ± 4.7	*F*, 0.910	0.405
AST (IU/L)	57.5 ± 4.6	49.3 ± 4.4	51.5 ± 4.4	*F*, 0.910	0.405
AP (IU/L)	139.7 ± 10.4	128.3 ± 10.7	147.0 ± 10.1	*F*, 0.874	0.419
GGT (IU/L)	130.1 ± 17.9	124.8 ± 17.4	111.7 ± 17.5	*F*, 0.287	0.751
Glucose (mg/dL)	106.8 ± 4.7	108.1 ± 4.6	101.8 ± 4.6	*F*, 0.533	0.588
Creatinine (mg/dL)	0.76 ± 0.02	0.78 ± 0.02	0.75 ± 0.05	*F*, 0.284	0.753

*Note:* Values are given as mean ± SD.

**FIGURE 2:**
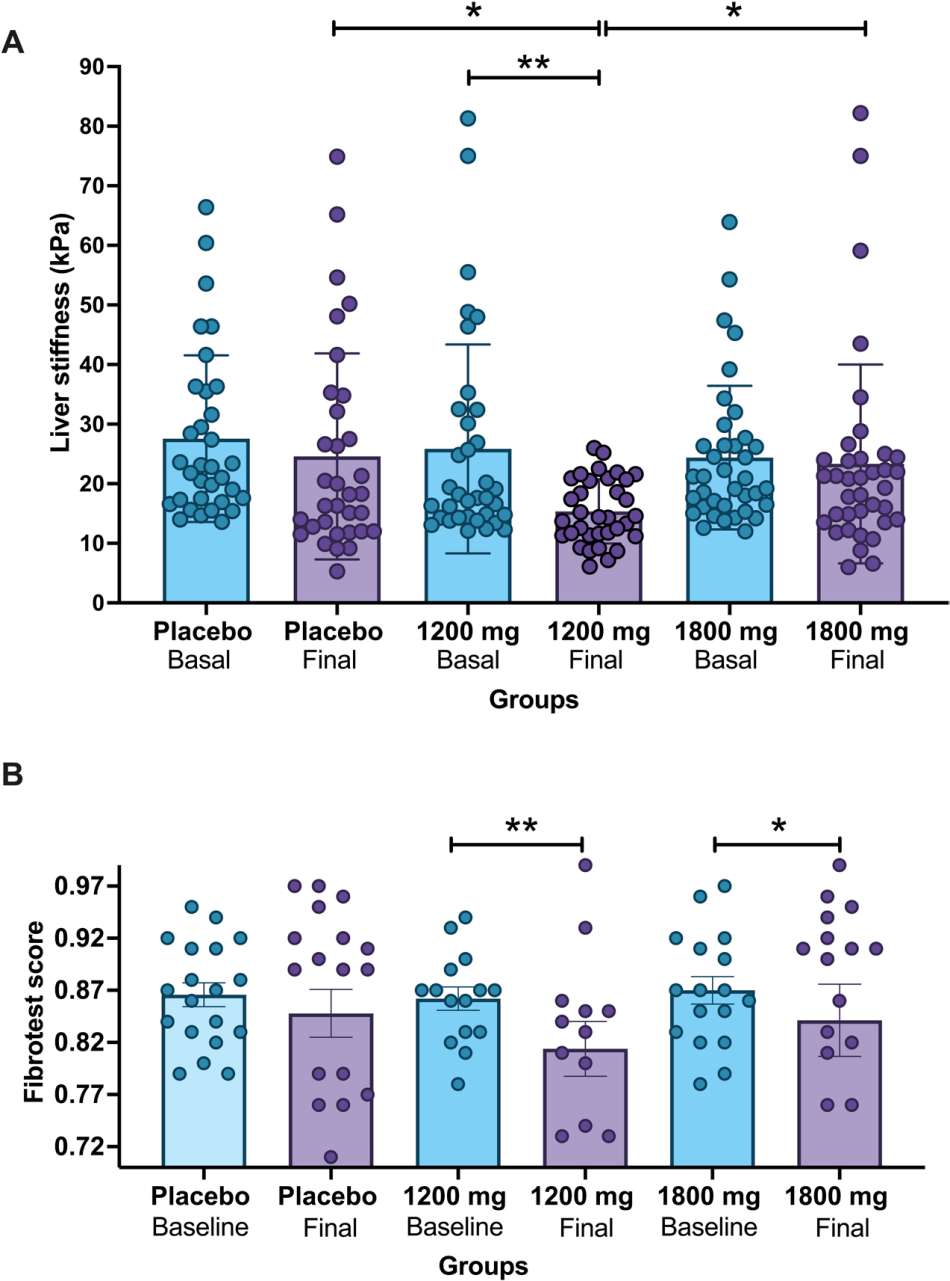
(A) Fibrosis evolution estimated by liver stiffness assessment (mean ± SE) shows a statiscally significant difference using the Mixed Model method (*p* < 0.001), for inter‐group analysis. Final values between the three groups showed also significant differences (placebo vs. 1200 mg, *p = 0.023; 1200 mg versus 1800 mg, *p = 0.012) in post hoc analysis with Bonferroni correction. Additionally, for intra‐group analisis only in the 1200 mg PR‐PFD group, compared to baseline values **p = 0.001, by Student's *t* test. (B) Fibrosis evolution estimated by Fibrotest assessment (mean ± SE). Statistical significance was analysed by Student's t‐test; **p = 0.001 in the 1200 mg, and *p = 0.045 in the 1800 mg PR‐PFD group, compared to baseline values.

Figure 2A shows the changes from the liver stiffness evaluations. Only patients in the 1200 mg PR‐PFD group obtained a statistically significant response when compared against placebo and 1800 mg PR‐PFD groups, in intergroup analysis (*p* < 0.001) by the Mixed Model methods and then confirmed by the ANCOVA method (*p* < 0.001, power = 0.971). In the ITT population (*n* = 165) the statistical significance was slightly lower (*p* < 0.046, power = 0.821).

Final values between the three groups also showed significant differences (placebo versus 1200 mg, *p* = 0.023; 1200 mg vs. 1800 mg, *p* = 0.012) in post hoc analysis with Bonferroni correction.

Table 3 describes the measurements of liver stiffness at baseline and at six, 12, 18, and 24 months of treatment in ITT population analysis (*n* = 165), showing a statistically significant response, evaluated by the mixed model for repeated measurements (*p* < 0.001). Relative changes are shown in supplemental files as Figure S1.

**TABLE 3:** Liver stiffness evolution from baseline to 24 months.

Liver stiffness assessment (kPa)	Placebo	1200 mg	1800 mg
At baseline	27.6 ± 2.4	24.2 ± 2.4	24.4 ± 2.3
At 6 months	27.9 ± 3.9	21.6 ± 4.2	24.9 ± 3.3
At 12 months	23.7 ± 2.4	17.7 ± 2.4	23.5 ± 2.3
At 18 months	26.6 ± 3.4	14.8 ± 3.3	24.0 ± 3.2
At 24 months	24.6 ± 2.4	15.4 ± 2.4	23.3 ± 2.3
Intragroup *p* values	0.402	0.001	0.654

*Note:* Values are given as mean ± SE. Global statistical significance was analysed by a mixed model of repeated measurements, *p* < 0.001 for intergroup analysis.

In addition, a significant decrease/increase in fibrosis was defined as a 50% change compared to the baseline kPa values. Patients who received 1200 mg of PR‐PFD had less FPP than patients who received placebo (2.9% vs. 18.2%, *χ* ^2^ = 4.709, *p* = 0.030), or 1800 mg of PR‐PFD (2.9% vs. 16.2%, j*χ* ^2^ = 4.045, *p* = 0.044), according to the probability test ratio. Additionally, patients receiving 1200 mg had higher FRP than placebo (25.7% vs. 12.1%, *χ* ^2^ = 2.078, *p* = 0.149), and than 1800 mg PR‐PFD (25.7% vs. 10.8%, *χ* ^2^ = 2.750, *p* = 0.097).

Liver fibrosis using FT also showed a slight but statistically significant reduction in PP (*n* = 105) and ITT population (*n* = 165) analysis, particularly in patients treated with 1200 or 1800 mg of PR‐PFD (Figure 2B). When evaluating FT values semiannually, according to the mixed model of repeated measurements, patients on placebo remained stable at their baseline values (0.87 ± 0.02) and at six months (0.87 ± 0.02), 12 months (0.84 ± 0.02), 18 months (0.82 ± 0.02) and 24 months (0.85 ± 0.02, *p* = 0.101); while patients on 1200 mg PR‐PFD had an intra‐group statistically significant reduction in the semi‐annual measurement from baseline (0.86 ± 0.02) to six months (0.85 ± 0.02), 12 months (0.81 ± 0.02), 18 months (0.80 ± 0.02), and 24 months (0.82 ± 0.02, *p* = 0.001). Furthermore, patients on 1800 mg PR‐PFD also had a slight but significant intra‐group reduction during treatment from baseline (0.87 ± 0.02) to six months (0.85 ± 0.02), 12 months (0.81 ± 0.02), 18 months (0.86 ± 0.02), and 24 months (0.84 ± 0.02, *p* = 0.045). No significant differences in inter‐group analysis were found using the Mixed Model method (*p* = 0.352). However, in the ANCOVA method a significant difference was found for intergroup analysis in the PP population (*n* = 105, *p* < 0.012, power = 0.848) but not in the ITT population.

The FIB‐4 scores were also significantly reduced, according to the mixed model for repeated measurements (*p* = 0.002), attaining internal significance only in the 1200 mg PR‐PFD group (4.66 ± 0.38 vs. 3.67 ± 0.45 units, *p* = 0.05). A moderate positive correlation was found between the independent values of the log_10_ scores for liver stiffness (kPa) and the FIB‐4 scores (*r* = 0.36, *p* < 0.0001).

Regarding biochemical evolution, Table 4 presents the calculated ratios between the final values with respect to the baseline values. The statistically significant increase in platelet (*p* < 0.007) and albumin (p < 0.01) values stands out, as well as the reduction in bilirubin (*p* < 0.01) and alkaline phosphatase values in the PR‐PFD 1200 mg group compared to the placebo group. Liver enzymes, ALT, and AST decreased in all three patient groups. It is notable that neither the patients taking 1200 mg PR‐PFD nor those taking 1800 mg for 24 months had elevated liver enzymes during the entire protocol. Furthermore, when baseline versus final results were analysed, only patients in the 1200 mg PR‐PFD group showed significantly decreased values (43.4 ± 3.8 vs. 31.3 ± 4.8 IU/L, *p* = 0.003).

**TABLE 4:** Relationship between final values at 24 months with respect to basal values.

Parameters	Placebo	1200 mg	1800 mg	*p*
Platelets (×10^3^)	1.02	1.19^a^	1.08	0.007
Total bilirubin (mg/dL)	1.15	0.72^b^	0.87	0.001
Albumin (mg/dL)	0.98	1.08^b^	1.01	0.001
Aspartate aminotransferase (IU/L)	0.83^c^	0.80^c^	0.92	0.002
Alanine aminotransferase (IU/L)	0.77^c^	0.72^c^	0.87	0.072
Alkaline phosphatase (IU/L)	1.00	0.78^a^	1.11	0.001
Body weight (kg)	1.03	0.98	1.00	0.848

*Note:* Values are given as rates. Statistical significance was analysed by ANOVA test.

^a^ Comparing 1200 mg PR‐PFD group against placebo and 1800 mg PR‐PFD groups.

^b^ Comparing 1200 mg PR‐PFD group against placebo.

^c^ Comparing placebo and 1200 mg PR‐PFD group against baseline values.

The evolution of the Child–Pugh and MELD scores shows that a significantly greater proportion of patients treated with 1200 mg PR‐PFD maintained a stable score (91%) compared with patients on placebo (73%, *p* = 0.040) and those in the 1800 mg population (72%, *p* = 0.020) using the likelihood ratio test. MELD scores were improved only in the 1200 mg PR‐PFD group (9.7 ± 0.32 vs. 9.0 ± 0.40, *p* = 0.022), compared to the placebo (9.9 ± 0.33 vs. 10.2 ± 0.42, *p* = 0.862) or 1800 mg PR‐PFD (9.5 ± 0.32 vs. 9.1 ± 0.39, *p* = 0.114). MELD profiles increased more frequently, without reaching statistical significance, in the placebo group (21%) than in the 1200 mg PR‐PFD group (9%, Xi^2^ = 2.209, *p* = 0.137) and decreased more frequently in the 1200 mg PR‐PFD group (31%) than in the placebo group (25%, Xi^2^ = 2.555, *p* = 0.110), according to the likelihood ratio test.

According to the visual analog scale, quality of life subjectively improved among all participants. However, based on a more robust scale, such as the EQ‐5D score, only patients treated with 1200 mg PR‐PFD had higher and statistically significant scores compared to the baseline (81.6 ± 2.1 vs. 88.1 ± 2.6, *p* = 0.004, data not shown). The fatigue rating scale scores improved slightly in the three groups, without statistical significance.

Based on the Baveno VII guidelines and the liver stiffness score, we found a significantly higher proportion of stable low scores (< 25 kPa) and decreasing scores (defined as the change of high rigidity values > 25 kPa to a low score < 25 kPa) in patients on 1200 mg PR‐PFD (91.2%) compared to those on placebo (60.6%, *χ* ^2^ = 9.115, *p* = 0.003) or 1800 mg of PR‐PFD (80%, *χ* ^2^ = 3.109, *p* = 0.078), using the likelihood ratio test and the PP population for analysis.

Regarding decompensation, 27 patients presented with at least one symptom of disease progression. However, a greater number of patients in the placebo group had complications of ascites compared to the 1200 mg and 1800 mg PR‐PFD groups, as shown in Table 5.

**TABLE 5:** Incidents and side effects detected in the total study population.

	Group 0 mg (*n* = 60)	Group 1200 mg (*n* = 60)	Group 1800 mg (*n* = 60)
Type of decompensation			
Variceal bleeding	3 (5.0%)	2 (3.3%)	0
Ascites	8 (13.3%)^a^	3 (5.0%)	1 (1.6%)
Encephalopathy	1 (1.6%)	2 (3.3%)	1 (1.6%)
Subtotals	12 (20.0%)	7 (11.7%)	2 (3,3%)
Event type			
Gastrointestinal disorders	35^a,b^	48	46
Nervous system disorders	19	23	20
Infections	20	16	14
Skin disorders	12	15	22^c,d^
Fatigue	3	4	7
Low back and tendinitis	8	2	6
Others	14	19	22
Total events	111	127	137
Serious AE^e^			
Serious	5	6	3
Non‐serious	106	121	134
AE Grade^e^			
Mild	64	64	64
Moderate	42	57	70
Severe	5	6	3
AE relation to study drug medication (Causality^e^)			
Certain	0	0	0
Probable	30	56	55
Possible	21	24	43
Improbable	60	47	39

*Note:* Type of decompensation: Values are given as percentages. Statistical significance was calculated by likelihood ratio test, *χ* ^2^ = 5.979, *p* = 0.016; ^a^Event type: Values are given as number of cases. Statistical significance was analysed by the likelihood ratio test. Comparing: placebo against 1200 mg, ^a^ *χ* ^2^ = 6.711, *p* = 0.010 or 1800 mg, ^b^ *χ* ^2^ = 4.644, *p* = 0.031; comparing 1800 mg group against placebo group, ^c^ *χ* ^2^ = 19.126, *p* = 0.0001; or against 1200 mg group, ^d^ *χ* ^2^ = 14.023, *p* = 0.0001. ^e^Based on criteria of NOM‐220‐SSA1‐2016.

Finally, a total of 375 AEs were identified during the 24 months of the study; the majority were considered mild and related to the GI tract (*n* = 129, 34.4%), nervous system disorders (*n* = 62, 16.5%), infections (*n* = 50, 13.3%), skin disorders (*n* = 49, 13.1%), or other conditions (*n* = 85, 22.7%). GI AEs were statistically higher in the 1200 mg (*n* = 48) and 1800 mg (*n* = 46) PR‐PFD treatment groups compared to the placebo‐treated (*n* = 35) group. The 1800 mg PR‐PFD dose (*n* = 22), but not the 1200 mg dose (*n* = 15), was associated with a greater number of cutaneous AEs compared with patients receiving a placebo (*n* = 12), as depicted in Table 5. Of the total skin events, 22 were considered photosensitivity reactions. Fatigue episodes [3, 4, 7] were more frequently seen in the 1800 mg PR‐PFD group than in the placebo or 1200 mg PR‐PFD (*p* = NS) and low back pain and tendinitis [2, 6, 8] were more frequently seen in the placebo group than in the 1200 or 1800 mg PR‐PFD groups (*p* = NS). The others section includes a mixed series of events with no significant differences between groups. A total of 14 serious adverse events (SAEs) were identified, with a similar distribution between groups (5 in placebo, 6 in 1200 mg, and 3 in 1800 mg PR‐PFD groups (*P* = NS)). Four cases of SARS COV2‐related pneumonia were detected (2 in placebo and 2 in 1800 mg). In the 1200 mg PR‐PFD group, one patient died during the study due to pneumonia and septic shock with multiple organ failure after hip replacement surgery. This was considered an unrelated SAE. Hepatocarcinoma, a liver‐related event, occurred in 4 patients (0 mg = 1; 1200 mg = 2; 1800 mg = 1) without a statistical difference between groups.

Table 6 depicts the plasma PFD levels in patients receiving PR‐PFD, including the same patients throughout the 9 visits showing significantly increased levels in 1800 mg versus 1200 mg PR‐PFD groups (Student's *t* test *p*‐values = 1.97, *p* = 0.0249).

**TABLE 6:** Mean plasma concentration of Pirfenidone (mean ± SE) in 1200 and 1800 mg PR‐PFD treated groups.

Sample evaluation time	PR‐PFD 1200 mg dose (*n* = 17)	PR‐PFD 1800 mg dose (*n* = 18)
Month‐1	7.57 ± 1.06	11.58 ± 1.37
Month‐3	9.94 ± 1.15	13.38 ± 1.40
Month‐6	9.38 ± 1.15	11.53 ± 1.37
Month‐9	8.77 ± 1.17	11.54 ± 1.95
Month‐12	8.71 ± 1.29	10.54 ± 2.03
Month‐15	9.04 ± 1.22	10.89 ± 2.10
Month‐18	10.56 ± 1.3	11.07 ± 1.41
Month‐21	9.45 ± 1.04	13.61 ± 2.50
Month‐24	8.19 ± 1.21	9.95 ± 1.41
Mean values	9.06 ± 0.38	11.61 ± 0.56*
Accumulated samples	153	162

* Student's *t* test *p*‐values = 1.97, *p* = 0.0249.

## 4 Discussion

To our knowledge, this clinical trial is the first to evaluate the efficacy and safety of PR‐PFD in patients with compensated cirrhosis. The main contribution of this trial was the significant reduction in liver stiffness and surrogate markers of liver fibrosis after 24 months of treatment in patients receiving an oral dose of 1200 mg per day of PR‐PFD. We previously showed three profiles of fibrosis evolution [9], and we now confirm that a fibrosis regression profile greater than 50% with respect to the baseline value of liver stiffness was observed more frequently in the PR‐PFD group. Other relevant findings are the significant improvement in platelet count, bilirubin, and albumin values commonly used as part of noninvasive biological tests for suspecting the presence of ALF [26].

This ODISEA study provides useful information on the long‐term safety of this drug, once considered by the FDA as potentially dangerous for patients with cirrhosis [27]. In fact, all patients, including those receiving placebo and both doses of PR‐PFD, showed a slight but significant reduction in ALT and AST serum levels (as well as ALP levels), administered continuously over 24 months, demonstrating anti‐inflammatory properties and a good safety profile in patients with compensated liver cirrhosis.

Regarding the evolution of Child–Pugh and MELD scores, a significantly higher proportion of patients treated with PR‐PFD maintained a stable Child–Pugh score or achieved a reduction in the MELD score, compared to the placebo group. Furthermore, we found a higher proportion of scores lower than 25 kPa in the estimation of liver stiffness, in patients receiving 1200 mg PR‐PFD than in placebo‐receiving patients, a parameter considered a predictor of the risk of variceal haemorrhage, according to Baveno VII [28]. Hepatic decompensation occurred in 27/180 (15%) patients. A significantly greater number of patients in the placebo group were complicated by ascites compared to the PR‐PFD groups.

In terms of quality of life, all participants reported subjective improvement according to the visual analog scale. However, when using a more robust scale, such as the EQ‐5D score, only patients treated with 1200 mg PR‐PFD scored significantly higher statistically compared to the baseline.

Finally, in relation to the safety of participating patients, a total of 375 AEs were identified during the study, most of them considered mild and related to the GI tract or skin, as seen in patients with IPF, a population where the chronic use and safety of PFD has been widely reported [7, 29]. Recommended strategies to mitigate adverse effects and ensure safe and continued use of pirfenidone are close patient monitoring, dose adjustments, and supportive therapies, including diet modification, antiemetics, and skin care [30, 31].

In Mexico, PFD has been developed and introduced to the market as a prolonged‐release formulation (PR‐PFD) that can be administered every 12 h instead of the usual 8‐h administration dose for the standard‐release PFD. Importantly, the pharmacokinetic profile of PR‐PFD revealed less fluctuation in Cmax and Cmin, which may facilitate the tolerability and efficacy of the drug formulation [32].

Explaining the lack of efficacy of new compounds metabolised by cytochrome P450 in patients with liver cirrhosis poses several complexities. In cirrhosis, the liver's ability to perform metabolic functions is significantly impaired due to the loss of functional hepatocytes and alterations in liver architecture. This impairment often results in diminished activity of microsomal enzymes, crucial for the metabolism of many drugs. Consequently, drugs that rely heavily on cytochrome P450 for clearance may exhibit altered pharmacokinetics, leading to therapeutic failures or unexpected toxicity [33]. The most common site of drug metabolism is the liver. At low doses, drugs are usually metabolised in first‐order kinetics. However, at higher doses, the enzymes may become saturated, shifting to zero‐order kinetics, and altering the dose‐effect relationship. This is most evident when the liver is damaged because changes in the pharmacokinetics and pharmacodynamic drug properties are common in many chronic liver diseases.

The lack of efficacy of the 1800 mg dose of PR‐PFD observed in this study can be explained by the cytochrome P450 (CYPs) saturation phenomenon, the site where PR‐PFD is metabolised. The CYPs are the most important enzymes in the oxidative metabolism of hydrophobic drugs such as PFD, a synthetic small‐molecule derivative of pyridone [5‐methyl‐1‐phenyl‐2(1H)‐pyridone]. Because of its size and hydrophobic nature, PFD is able to diffuse freely across cell membranes without using a receptor within the liver or other target tissues [6]. It has been reported that the versatility of CYPs enzymes may result in some unusual kinetic properties, stemming from the simultaneous interaction of multiple substrates with the CYP active site. According to our findings in the 1800 mg PR‐PFD dose, further studies will be needed to evaluate interactions between substrates or the possibility of competitive or noncompetitive inhibition, mixed inhibition, partial inhibition, activation, and activation followed by inhibition, as previously described [34]. A pharmacokinetic study of PR‐PFD performed by our group showed that the area under the curve and Cmax significantly increased in patients with cirrhosis compared to subjects without cirrhosis [19]. In this study, we found significantly higher plasma PFD levels in 1800 mg than in the 1200 mg PR‐PFD group throughout the study. This greater exposure of the drug in cirrhosis, a condition which entails a reduction in liver mass and the ability to metabolise certain medications, could explain our findings.

We do not have a complete mechanistic understanding of the lack of efficacy at the higher dose, but we hypothesise that chronic overload with PFD is related to the loss of efficacy of a compound that theoretically should have a positive, antifibrotic, dose‐related effect.

To better understand the relationship between exposure and outcome, and the possibility of a non‐linear relationship between dose and effect observed in our study, we recommend that future studies with PR‐PFD should consider evaluating the efficacy of a lower dose range at least in patients with cirrhosis [35].

We are aware of some limitations of our study related to the lack of liver biopsies for evaluating variations in the degree of fibrosis. Liver biopsy was long considered the gold standard, but it is associated with several limitations [36]. Furthermore, due to spatial heterogeneity in the distribution and the density of scar tissue, variability and sampling errors are induced. In addition, high interobserver variability may affect the accuracy of the assessment. Finally, it is possible that the dynamic nature of liver fibrosis cannot be captured in such a small, random sample of tissue obtained in a single biopsy [37, 38].

The ODISEA study opted for the evaluation of liver fibrosis using NITs, such as TE, and serum biomarker assays, which estimate liver stiffness and provide accurate and reliable assessments of liver fibrosis behaviour without the need for invasive procedures [26, 39, 40, 41]. Although they also have their own limitations, they offer greater patient comfort, reduce the risk of complications, and allow longitudinal monitoring of disease progression [42].

Other promising drugs, such as obethicolic acid (OCA) [43], cenicriviroc (CVC) [44], and resmetiron [45] have been evaluated in patients with MASLD and various degrees of liver fibrosis (from F1 to F3). Although the authors of these studies found significant improvements in metabolic activity, only a slight improvement in liver fibrosis was detected. In the OCA trial, 23% of patients receiving OCA experienced at least a 1‐stage improvement in fibrosis compared to 12% in the placebo group. The authors claimed their findings highlight the importance of non‐invasive evaluation techniques in monitoring patient progress without the need for invasive liver biopsies [43]. The CVC trial did not show significant efficacy in improving liver fibrosis compared to the placebo. The rates of fibrosis improvement in the CVC group were not markedly different from those observed in the placebo group (22.3% vs. 25.6%; *p* = 0.21) [44]. In the Resmetiron trial evaluating patients with F1 up to F3, 24.2%–25.9% of patients achieved at least a one‐stage improvement in fibrosis without worsening of NASH, compared to 10% in the placebo group (*p* < 0.001). However, patients with cirrhosis (F4) were not included [45]. According to recent EASL clinical practice guidelines, no MASH‐targeted pharmacotherapy can currently be recommended for the cirrhotic stage [46]. In this context, it is important to clarify that the ODISEA trial included patients with compensated liver cirrhosis (F4) of mixed aetiology and demonstrated an improvement in fibrosis in at least one‐third of the patients who received the 1200 mg dose of PR‐PFD.

Heterogeneity of the cause of cirrhosis can be another issue. However, no statistically significant differences in aetiology were found. The behaviour of fibrosis can vary depending on the aetiology; However, we consider that this limitation could be overcome with the wealth of information of our target population which represents the transition from HCV to MASLD as the predominant causes in the last decades in the world and Mexico [4, 47].

Focusing on a single aetiology would allow for more homogeneous patient populations. By isolating a specific cause, researchers could better understand the drug's mechanism of action and ensure that outcomes are attributable to treatment rather than confounding factors inherent in a heterogeneous population. We therefore recommend that future studies evaluate the efficacy of PR‐PFD in clearly delineated patient populations.

We are aware that the aetiology of cirrhosis can induce variants in the formation of the matrisome and even CSPH can reflect different clinical evolution scenarios [48]. On the other hand, it would be ideal that patients with chronic hepatic disease never develop cirrhosis, or that approaches to fibrosis regression are initiated before the patient reaches the point of no return. To access this Gold Standard, several authors have proposed that in addition to treating the aetiology, future treatments for ALF should include matrisome reversal, TIMMP reduction, and MMP enhancement, allowing the stimulation of specialised macrophages in fibrosis regression, where TGFb‐1 inhibition plays a pivotal role, effects previously described by PFD in experimental liver cirrhosis by a Mexican group [10, 11, 12]. Furthermore, Rodríguez‐Sanabria et al. [49], have reported that H3K9me3 demethylation by JMJD2B is regulated by PFD, resulting in an improvement of NASH or slowing down experimental HCC development by controlling DNA methylation [50].

We agree with Friedman and Pinzani that there is a need to use serum markers or frequent measurements of liver stiffness in clinical practice that accurately indicate when fibrosis is regressing [48]. We therefore hope that our findings may be useful in better understanding the biology of fibrosis regression.

Another partial limitation of our study relates to patient dropouts (*n* = 5), which were partially related to the COVID‐19 pandemic, with a similar distribution between the groups. We also had inclusion errors that forced a reduction in our final study population (*n* = 165) which fortunately was higher than that obtained in the calculation of the study size population (*n* = 105).

We conclude that PR‐PFD demonstrated statistically significant efficacy in reducing noninvasive markers of liver fibrosis and improving biological tests associated with liver function (albumin, bilirubin, Child–Pugh, and MELD scores). It also provides a better safety profile because of its association with a lower incidence of ascites and a better quality of life. Furthermore, our study contributes to providing evidence for the safety of long‐term use of PR‐PFD in patients with liver cirrhosis, as we observed a low incidence of AEs and a significant reduction in liver enzymes. More studies with more robust, homogeneous populations and other diagnostic markers are required to confirm the effectiveness of PR‐PFD against liver fibrosis, a condition that represents a great burden on global health.

## Author Contributions

A.T., L.C., I.M., R.M., S.M., J.R.A., J.L., J.Z.‐N., and M.E.I. were involved in population recruitment and the ambulatory care of the patients. F.G.‐D., L.H.‐H., P.C.‐P., L.C., L.T., and F.R.‐A. were involved in the ambulatory and nutritional care and carried out the capturing and integration of the database with all demographic, clinical, and biochemical data; G.T. participated in protocol design and planning sample size calculation, and conducted the biostatistical analysis. L.E.M.‐E. was involved in protocol design and planning, patient recruitment, and manuscript writing. J.L.P. led the multicenter group working with PR‐PFD, was involved in recruitment and ambulatory care, and oversaw the manuscript writing and style. All authors read and approved the final manuscript.

## Ethics Statement

The protocol was approved by local IRBs, and the Mexican Drugs Agency (COFEPRIS).

## Consent

All patients were requested to read and sign the Patient Consent Form previously approved by local Mexican Drugs Agency (COFEPRIS).

## Conflicts of Interest

The authors declare no conflicts of interest.

## Supporting information


Figure S1.

## Data Availability

The data that support the findings of this study are available from the corresponding author upon reasonable request.
