# Supplementary material for: Noninvasive Evaluation of Prolonged‐Release Pirfenidone in Compensated Liver Cirrhosis. ODISEA Study, a Randomised Trial
Source: Liver Int. 2025 May 22;45(6):e70131. doi: 10.1111/liv.70131 (PMC12097196; doi:10.1111/liv.70131)
Supplement: Supplementary file 1 — Figure S1. [file LIV-45-0-s001.docx]

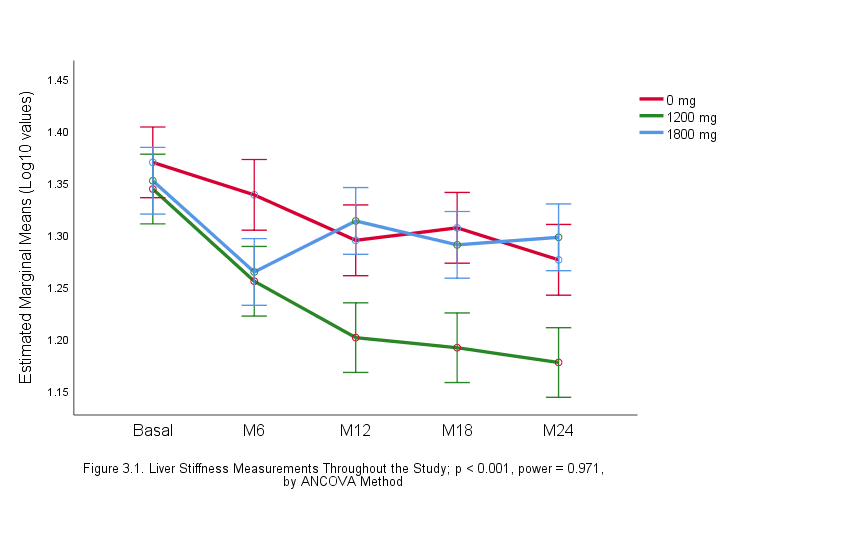


Supplementary figure 1. Liver stiffness measurements throughout the study; p = 0.001,

Power = 0.971, by ANCOVA method.
